# A three miRNAs signature predicts survival in cervical cancer using bioinformatics analysis

**DOI:** 10.1038/s41598-017-06032-2

**Published:** 2017-07-17

**Authors:** Bin Liang, Yunhui Li, Tianjiao Wang

**Affiliations:** 1 Department of Bioinformatics, Key Laboratory of Cell Biology, Ministry of Public Health and Key Laboratory of Medical Cell Biology, Ministry of Education, College of Basic Medical Science, China Medical University, Shenyang, China; 2 Department of Clinical Laboratory, No. 202 Hospital of PLA, Shenyang, China

## Abstract

Growing evidences showed that a large number of miRNAs were abnormally expressed in cervical cancer tissues and played irreplaceable roles in tumorigenesis, progression and metastasis. The aim of the present study was to identify the differential miRNAs expression between cervical cancer and normal cervical tissues by analyzing the high-throughput miRNA data downloaded from TCGA database. Additionally, we evaluated the prognostic values of the differentially expressed miRNAs and constructed a three-miRNA signature that could effectively predict patient survival. According to the cut-off criteria (*P* < 0.05 and |log_2_FC| > 2.0), a total of 78 differentially expressed miRNAs were identified between cervical cancer tissues and matched normal tissues, including 37 up-regulated miRNAs and 41 down-regulated miRNAs. The Kaplan-Meier survival method revealed the prognostic function of the three miRNAs (miRNA-145, miRNA-200c, and miRNA-218-1). Univariate and multivariate Cox regression analysis showed that the three-miRNA signature was an independent prognostic factor in cervical cancer. The functional enrichment analysis suggested that the target genes of three miRNAs may be involved in various pathways related to cancer, including MAPK, AMPK, focal adhesion, cGMP-PKG, wnt, and mTOR signaling pathway. Taken together, the present study suggested that three-miRNA signature could be used as a prognostic marker in cervical cancer.

## Introduction

According to the world cancer statistics, cervical cancer is the fourth most common cancer affecting women globally and the second most common cancer in developing areas^1, 2^, with an estimated global incidence of 530,000 new cases and 270,000 deaths annually^3^. The preventive vaccination and organized screening programs are critical in identifying the cervical cancer before it enters advanced stages. Moreover, the treatments are often less effective in advanced stages compared with early interventions^4^. Thus, understanding of the molecular mechanisms of cervical cancer development and identification of novel biomarkers are required for the early detection and treatment of cervical cancer.

MicroRNAs (miRNAs), a key component of the noncoding RNA family, are approximately 18–25 nucleotides that involved in the post-transcriptional regulation of gene expression^5^. It has been shown that miRNAs are aberrantly expressed in various types of malignancies and function either as oncogenes or tumor suppressors^6^. Accumulating evidence has demonstrated that miRNAs regulated various carcinogenesis processes including cell maturation^7^, cell proliferation^8^, migration, invasion^8^, autophagy^8^, apoptosis^9^, and metastasis^10^. Therefore, miRNAs have a large potential to serve as promising markers in the diagnosis, prognosis, and personalized targeted therapies.

Although a number of miRNAs have been identified in predicting the clinical outcome in cervical cancer, there exists inconsistence in previous studies. This may due to the small sample size, heterogeneous histological subtype, different detection platforms, and various data processing methods. The Cancer Genome Atlas Project (TCGA) is a National Cancer Institute effort to profile at least 20 different tumor types using genomic platforms and to make raw and processed data available to all researchers^11^. The TCGA released a large number of miRNA sequencing data for cervical cancer patients. The aim of the present study was to identify the differential miRNAs expression between cervical cancer tissues and matched normal cervical tissues by analyzing the high-throughput miRNA data downloaded from TCGA database. Additionally, we evaluated the prognostic value of the differential expressed miRNAs and constructed a three-miRNA signature that could effectively predict patient survival. Furthermore, we analyzed the pathway and function of the target genes of three miRNAs, which may provide novel insights into understanding the underlying molecular mechanism of cervical cancer.

## Results

### Identification of differentially expressed miRNAs in cervical cancer

**Figure 1:**
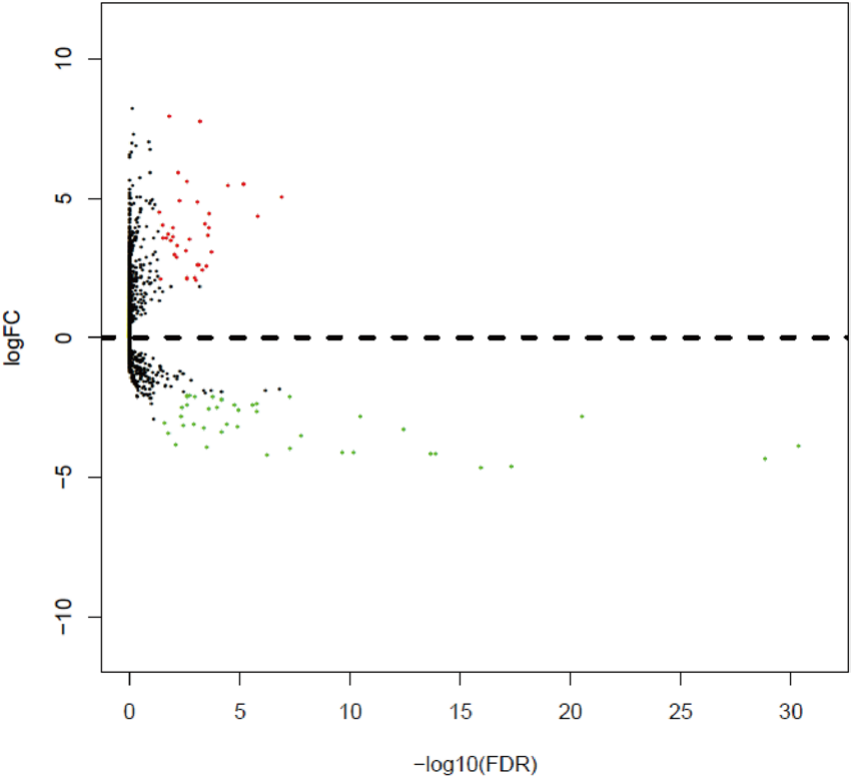
Volcano plot of differentially expressed miRNAs. The red dot represents up-regulated miRNA, and green dot represents down-regulated miRNA.

In the present study, a total of 254 samples were enrolled in this study, including 251 cervical cancer tissues and 3 matched normal tissues. The detailed clinical characteristics include diagnosis at age, metastasis, lymph node status, stage, T stage, histological type, pregnancy numbers, and smoking history category (Table 1). According to the cut-off criteria (*P* < 0.05 and |log_2_FC| > 2.0), a total of 78 differentially expressed miRNAs were identified between cervical cancer tissues and matched normal tissues, including 37 up-regulated and 41 down-regulated miRNAs (Table S1). In order to prove the *P* value and |log_2_FC| whether conform to logic with different test, we present the result as Volcano plot (Fig. 1). Unsupervised hierarchic cluster analysis revealed that cervical cancer tissues could be distinguished from normal tissues based on differentially expressed miRNAs patterns (Figure S1).

**Table 1:** Clinical characteristics of cervical cancer patients.

Variables	Case, n (%)
Age at diagnosis	
<60	196 (78.1%)
≥60	55 (21.9%)
Metastasis	
M0	92 (36.7%)
M1	9 (3.6%)
MX	109 (43.4%)
NA	41 (16.3%)
Lymph node status	
N0	105 (41.8%)
N1-2	51 (20.3%)
NX	57 (22.7%)
NA	38 (15.1%)
Stage	
I + II	191 (76.1%)
III + IV	54 (21.5%)
NA	6 (2.4%)
T stage	
T1 + T2	169 (67.3%)
T3 + T4	29 (11.6%)
TX	15 (6.0)
NA	38 (15.1%)
Histologic Type	
Squamous Cell Carcinoma	209 (83.2%)
Adenocarcinoma	42 (16.8%)
Number of pregnancies	
≤5	188 (74.9%)
>5	31 (12.4%)
NA	32 (12.7%)
Smoking History Category	
<3	174 (69.3%)
≥3	43 (17.1%)
NA	34 (13.5%)

NA, non available.

### Identification of three miRNAs associated with OS in cervical cancer

**Figure 2:**
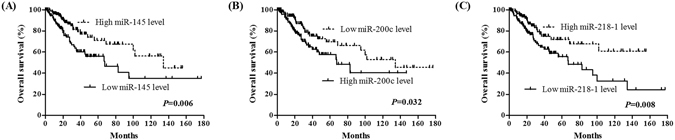
Three miRNAs were associated with overall survival in cervical cancer patients by using Kaplan-Meier curve and Log-rank test. The patients were stratified into high level group and low level group according to median of each miRNA. (**A**) miR-145; (**B**) miR-200c; and (**C**) miR-218-1.

To identify the miRNAs which would be potentially associated with overall survival of cervical cancer patients, we evaluated the association between miRNAs expression and patients’ survival using Kaplan-Meier curve and Log-rank test. The results showed that one miRNA (miR-200c) was negatively correlated with overall survival (OS), and two miRNAs (miR-145 and miR-218-1) were positively related to OS (Fig. 2). The association between three miRNAs and clinical features was evaluated in cervical cancer patients (Table 2). The results showed that miR-145 was significantly associated with metastasis (*P* = 0.033) and T stage (*P* < 0.001); miR-218-1 was associated with stage (*P* = 0.004), T stage (*P* = 0.001), and histological type (*P* < 0.001). No significant difference was found between miR-200c and clinical features (*P* > 0.05).

**Table 2:** Association of three miRNAs and clinical features.

Variables	Numbers	miR-145	*P* value	miR-200c	*P* value	miR-218-1	*P* value
Age at diagnosis							
<60	196	12.20 ± 1.04	0.963	15.46 ± 0.88	0.495	5.86 ± 1.31	0.392
≥60	55	12.21 ± 1.51		15.55 ± 0.81		6.03 ± 1.23	
Metastasis							
M0	92	12.28 ± 1.16	0.033	15.50 ± 0.88	0.456	5.89 ± 1.27	0.774
M1	9	11.39 ± 1.39		15.74 ± 1.27		6.02 ± 1.90	
Lymph node status							
N0	105	12.31 ± 1.17	0.882	15.45 ± 0.89	0.646	6.09 ± 1.38	0.970
N1-2	51	12.33 ± 1.04		15.51 ± 0.75		6.10 ± 1.13	
Stage							
I + II	191	12.25 ± 1.13	0.088	15.49 ± 0.83	0.915	6.02 ± 1.31	0.004
III + IV	54	11.94 ± 1.91		15.47 ± 1.01		5.45 ± 1.26	
T stage							
T1 + T2	169	12.26 ± 1.15	<0.001	15.51 ± 0.84	0.698	6.06 ± 1.31	0.001
T3 + T4	29	11.40 ± 1.12		15.58 ± 1.07		5.21 ± 1.11	
Histologic Type							
Squamous Cell Carcinoma	209	12.21 ± 1.18	0.679	15.44 ± 0.86	0.082	5.76 ± 1.18	<0.001
Adenocarcinoma	42	12.13 ± 1.01		15.69 ± 0.86		6.57 ± 1.68	
Number of pregnancies							
≤5	188	12.20 ± 1.10	0.387	15.51 ± 0.84	0.353	5.94 ± 1.31	0.554
>5	31	12.00 ± 1.53		15.36 ± 0.91		5.79 ± 1.30	
SmokingHistory Category							
<3	174	12.19 ± 1.15	0.978	15.49 ± 0.84	0.996	5.95 ± 1.33	0.453
≥3	43	12.19 ± 1.11		15.41 ± 1.04		5.79 ± 1.24	

### Prognostic value of three miRNAs signature risk score in cervical cancer

**Figure 3:**
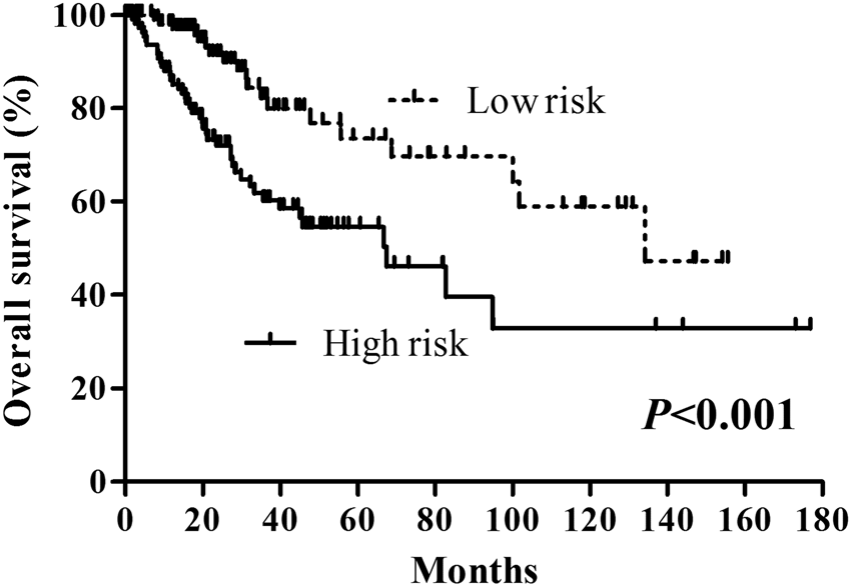
Kaplan-Meier curve for the three-miRNA signature in cervical cancer patients. The patients were stratified into high risk group and low risk group based on median.

We constructed a prognostic signature by integrating the expression profiles of three miRNAs and corresponding estimated regression coefficient. Then, we calculated a risk score for each patient, and ranked them according to increased score. Thus, a total of 251 patients were classified into a high risk group (n = 125) and a low risk group (n = 126) according to the median risk score. Survival analysis was performed using the Kaplan-Meier method with a Log-rank statistical test. The result showed that patients in high risk group have significantly worse OS than patients in low risk group (*P* < 0.001, Fig. 3).

Taking into account the following clinical features: age, metastasis, lymph node status, stage, T stage, histological type, pregnancy number, and smoking history category, univariate and multivariate Cox regression analysis were used to test the effect of the three-miRNA signature (high risk vs. low risk) on OS. In univariate analysis, age (HR = 0.562, *P* = 0.037), lymph node status (HR = 2.567, *P* = 0.010), stage (HR = 2.511, *P* = 0.001), T stage (HR = 4.640, *P* < 0.001), and three-miRNA signature (HR = 2.574, *P* < 0.001) were associated with OS in cervical cancer patients. In multivariate analysis, the three-miRNA signature (HR = 2.183, *P* = 0.028) was showed to be an independent prognostic factor in cervical cancer patients (Table 3).

**Table 3:** Univariate and multivariate Cox regression analysis in CC patients.

	Univariate analysis		Multivariate analysis	
	HR (95% CI)	*P* value	HR (95% CI)	*P* value
Age (≥60 vs. <60)	0.562 (0.327–0.967)	0.037		
Mestasis (M1 vs. M0)	2.355 (0.687–8.073)	0.173		
Lymph node status (N1–2 vs. N0)	2.567 (1.249–5.277)	0.010		
Clinical stage (III+IV vs. I + II)	2.511 (1.481–4.257)	0.001		
T stage (T3+T4 vs. T1+T2)	4.640 (2.328–9.245)	<0.001	3.876 (1.818–8.264)	<0.001
Histology type (SCC vs. Adenocarcinoma)	0.802 (0.381–1.690)	0.562		
Pregnancy (>5 vs. ≤5)	1.692 (0.873–3.280)	0.120		
Smoking history category (≥3 vs. <3)	0.739 (0.371–1.470)	0.387		
Three-miRNA signature (high risk vs. low risk)	2.574 (1.493–4.435)	0.001	2.183 (1.110–5.128)	0.028

### Target prediction and function analysis

**Figure 4:**
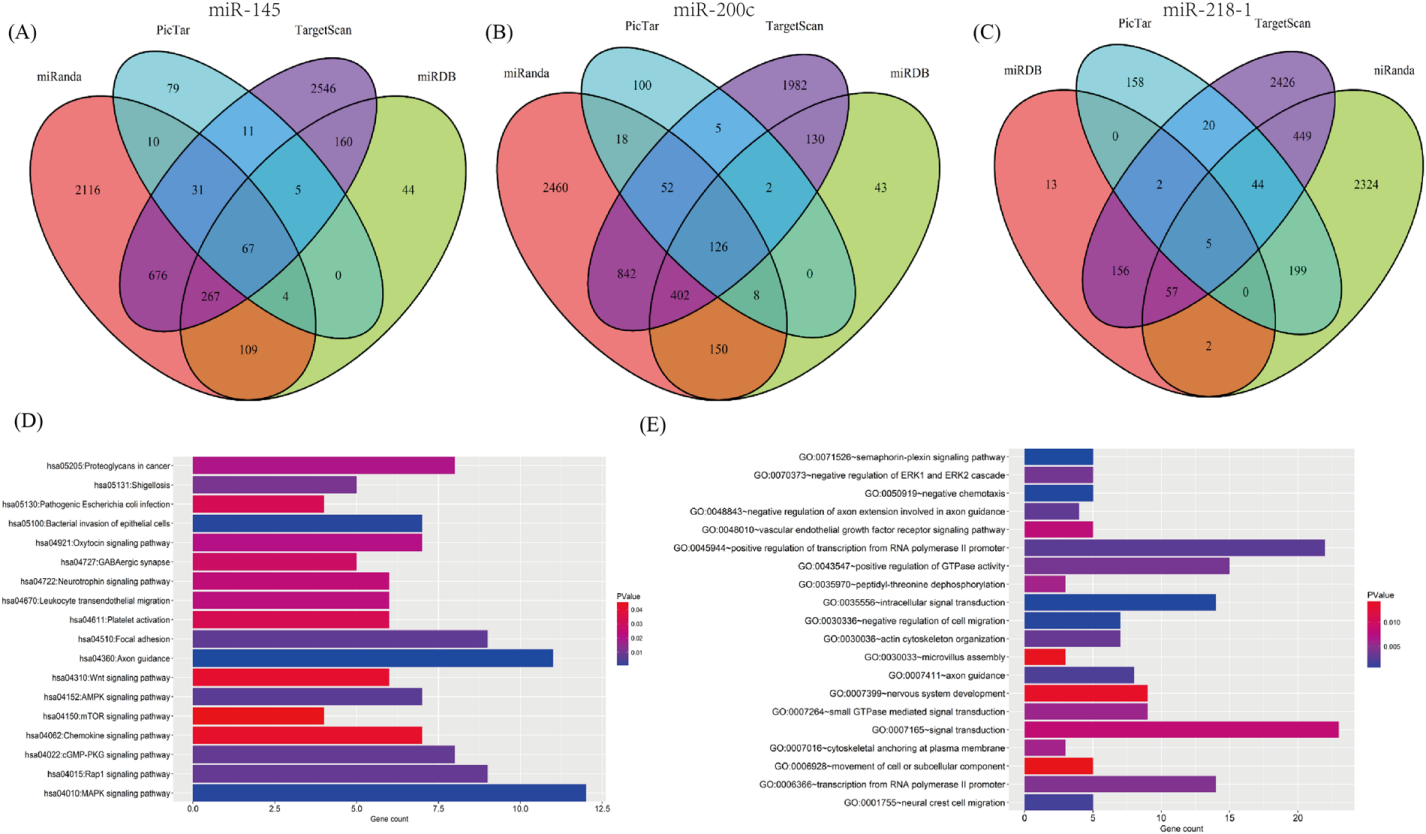
The target gene prediction and function analysis. The overlapping target genes were predicted using TargetScan, miRDB, PicTar, and miRanda online analysis tools. (**A**) miRNA-145; (**B**) miRNA-200c; (**C**) miR-218-1; (**D**) the significant enriched KEGG pathways of target genes; (**E**) the significant enriched GO biological processes of target genes.

The target genes of three miRNAs (miR-145, miR-200c, and miR-218-1) were predicted using TargetScan, miRDB, PicTar, and miRanda online analysis tools. A total of 67 overlapping genes of miR-145, 126 overlapping genes of miR-200c, and 5 overlapping genes of miR-218-1 were identified (Fig. 4). Then, enrichment analysis was performed to elucidate the biological function of consensus target genes. The KEGG pathways were significantly enriched in MAPK signaling pathway, AMPK signaling pathway, focal adhesion, cGMP-PKG signaling pathway, wnt signaling pathway, and mTOR signaling pathway. In addition, the GO biological process (BP) terms were mainly enriched in signal transduction, regulation of cell migration, and regulation of transcription.

## Discussion

With the introduction of vaccination and screening programs, the incidence of mortality associated with cervical cancer in developed areas have dramatically declined in recent decades, but the mortality in developing countries remains high, up to 87% of cervical cancer deaths^12–15^. The cervical cancer patient prognosis would be improved considerably if tumor behavior could be predicted reliably at the time of initial diagnosis. Therefore, understanding the molecular mechanisms of cervical cancer development and identification of novel biomarkers are needed. In the present study, a total of 78 differentially expressed miRNAs were identified, and three of them were associated with overall survival in cervical cancer patients. The three-miRNA (miR-145, miR-200c, and miR-218-1) signature was established and was identified to be an independent prognostic factor for cervical cancer patients. Moreover, we screened the target genes of these three miRNAs, and predicted the enrichment pathways and biological functions of target genes using bioinformatics methods.

In the last decade, MiRNAs, as the master modulators of multiple biological and pathological processes, are a hot research topic in the field of cancer development. Mounting evidence has demonstrated that miRNAs established a complex combinatorial system of gene expression and pathway regulation, as well as prognostic indicators and therapeutic targets in different cancers, including cervical cancer^16, 17^. Previous studies have demonstrated that many miRNAs are crucial for the initiation, progression and metastasis of cervical cancer by regulating various processes, including cancer cell proliferation, differentiation, apoptosis, adhesion, cell cycle arrest, migration and invasion^18^. To date, several studies had identified a number of miRNAs with prognostic values, such as miR-155^19^, miR-425-5p^20^, miR-196a^21^, miR-503^22^, miR-26b^23^, miR-335^24^, miR-3^25^, miR-215^26^, miR-224^27^, and so on. However, previous studies were based on small sample size, sample types, different detection platforms, various assay methods, and relatively limited numbers of miRNAs. In the present study, we analyzed high-throughput data, and identified that two down-regulated miRNAs (miR-145and miR-218-1) and one up-regulated miRNA (miR-200c) were associated with clinical outcome of cervical cancer patients. Zhou X, *et al*. reported miRNA-145 inhibits tumorigenesis and invasion of cervical cancer stem cells by inducing cancer stem cell (CSC) differentiation through down-regulation of the stem cell transcription factors that maintain CSC pluripotency^28^. Sathyanarayanan A, *et al*. suggested that miRNA-145 modulates epithelial-mesenchymal transition (EMT) and suppresses proliferation, migration and invasion by targeting SIP1 in human cervical cancer cells^29^. Previous article has also reported human papillomaviruses (HPV) oncoproteins E6 and E7 could suppress miR-145 expression^30^. Moreover, miR-218, as a tumor suppressor, was strongly down-expressed and related to proliferation, apoptosis and invasion in cervical cancer^31^. Yamamoto N, *et al*. demonstrated that miR-218, acting as a tumor suppressor in cervical cancer, inhibited cancer cell migration and invasion by targeting focal adhesion pathways in cervical squamous cell carcinoma^32^. In addition, HPV16 E6 promoted EMT and invasion in cervical cancer via the repression of miR-218, while miR-218 inhibited EMT and invasion in cervical cancer by targeting Scm-like with four MBT domains 1 (SFMBT1) and defective in cullin neddylation 1, domain containing 1 (DCUN1D1)^33^. MiR-200c, a member of miR-200 family, located on chromosome 12p13^34^. MiR-200c was confirmed to be down-regulated in human breast cancer stem cells, and up-regulated in ovarian cancer, lung cancer, gastric cancer, pancreatic cancer, colorectal cancer, and gastric cancer^35^, suggesting its complexity role in cancer as it can act either as oncogene or tumor suppressor depending on the origin of cancer. Our results showed that miR-200c was up-regulated in cervical cancer, and may be as an oncogene in development of cervical cancer. Furthermore, miR-145 was significantly associated with metastasis and T stage; miR-218-1 was associated with stage, T stage, and histological type, indicating miR-145 and miR-218-1 were involved in the progression of cervical cancer. But, no significant difference was found between miR-200c and clinical features. TCGA database does not provide completed cell differentiation (Grade), HPV infection, CIN stage, FIGO stage, and so on. Maybe, miRNA-200c was related to other factors. The future study will focus on this point, and investigate the function of miRNA-200c in cervical cancer.

In the present study, we found that miR-145, miR-200c, and miR-218-1 were differentially expressed, and significantly associated with overall survival in cervical cancer patients. While efficacy of a single marker was limited, multi-markers based model may provide more powerful information for the prognosis prediction of patients. We constructed three-miRNA signature, and the results suggested that the three-miRNA signature (high risk and low risk) predicted survival well, and was an independent prognostic factor in cervical cancer.

To gain a deep insight into the molecular functions of three miRNAs, we predicted the target genes and analyzed the related pathways and GO annotations. Abnormal signaling pathways play crucial roles in the pathogenesis and progression of cervical cancer. We found that three miRNAs could regulate several key signaling pathways, including MAPK signaling pathway, AMPK signaling pathway, focal adhesion, cGMP-PKG signaling pathway, wnt signaling pathway, and mTOR signaling pathway. Accumulating evidence has demonstrated that activation of MAPK signaling pathway is important in cervical cancer progression, invasion, and metastasis^36^. Yung M. M., *et al*. reported that activation of AMP-activated protein kinase (AMPK), a metabolic sensor, hampers cervical cancer cell growth through blocking the Wnt/β-catenin signaling activity^37^. The transformation of HPV expressing human keratinocytes requires activation of the Wnt pathway and that this activation may serve as a screening tool in HPV-positive populations to detect malignant progression^38^. Moreover, it has been well established that the PI3K/Akt/mTOR signaling pathway plays a crucial role in cervical cancer development^39^, and inhibition of mTOR kinase activity suppress tumor growth^40^. Therefore, further molecular investigations are needed to confirm these predictions, and it can provide new therapeutic interventions in cervical cancer.

Taken together, we identified three-miRNA signature as a potential prognostic predictor for cervical cancer patients. Further studies are needed to validate our findings in large sample size, and further function investigation are also required to explore the molecular mechanism of these miRNAs in cervical cancer progression.

## Materials and Methods

### Data processing

The raw sequencing data and clinical information were downloaded from TCGA database (https://cancergenome.nih.gov/). The inclusion criteria were set as follows: (1) the sample with both miRNA sequencing data and clinical information; (2) the sample with prognosis information. Finally, a total of 254 samples were enrolled in this study, including 251 cervical cancer tissues and 3 matched normal tissues. The detailed clinical characteristics and differentially expressed miRNAs were list in Table S2. The miRNA sequencing data were processed using R language package. The differentially expressed miRNAs between cervical cancer and normal tissues were analyzed by limma package in R. The fold changes (FCs) in the expression of individual miRNA were calculated and differentially expressed miRNAs with log_2_|FC| > 2.0 and *P* < 0.05 were considered to be significant.

### Association of differentially expressed miRNAs and patient prognosis

The differentially expressed miRNA profiles were normalized by log2 transformed. The prognostic value of each differentially expressed miRNA was evaluated using Kaplan-Meier curve and Log-rank method. The miRNAs that were significantly associated with overall survival were identified as prognostic miRNAs, and then subjected to a binary logistic regression analysis. Subsequently, a prognostic miRNA signature was constructed, and the miRNA signature could calculate a risk score for each cervical cancer patient. With the miRNA signature, cervical cancer patients were classified into high risk and low risk groups using the median risk score. Then, the differences in patients’ survival between the high risk group and low risk group were evaluated by Kaplan-Meier method.

### The target gene prediction of prognostic miRNA signature

The target genes of prognostic miRNAs were predicted using TargetScan (http://www.targetscan.org/), miRDB (http://www.mirdb.org/miRDB/), PicTar (http://pictar.mdc-berlin.de/), and miRanda (http://www.microrna.org/) online analysis tools. To further enhance the bioinformatics analysis reliability, the overlapping target genes were identified using Venn diagram. Then, the overlapping genes were analyzed by The Database for Annotation, Visualization and Integrated Discovery (DAVID) bioinformatics tool (https://david.ncifcrf.gov/). DAVID is a web-based online bioinformatics resource that aims to provide a comprehensive set of functional annotation tools for the investigators to understand the biological mechanisms associated with large lists of genes/proteins^41^. Gene Ontology (GO) and Kyoto Encyclopedia of Genes and Genomes (KEGG) pathway enrichment analyses were then performed for the target genes. The *P*-value < 0.05 and gene count ≥ 3 were set as the cut-off criteria.

### Statistical analysis

The data were expressed as mean ± standard deviation (SD). The expression levels of miRNAs in cervical cancer and matched normal tissues were analyzed by unpaired *t* test. The chi-square and *t* tests were performed to assess the relationship between miRNA expression and clinical features. Kaplan-Meier survival analysis and univariate/multivariate Cox proportional hazard regression analysis were carried out to compare each miRNA (low vs. high level) and prognostic miRNA signature (low vs. high risk). *P* value less than 0.05 was considered as statistical significant. The statistical analysis was performed using IBM SPSS Statistics software program version 22.0 (IBM Corp., NY, USA).

## Electronic supplementary material

Supplementary Information

Table S2
