# Supplementary material for: A three miRNAs signature predicts survival in cervical cancer using bioinformatics analysis
Source: Sci Rep. 2017 Jul 17;7:5624. doi: 10.1038/s41598-017-06032-2 (PMC5514022; doi:10.1038/s41598-017-06032-2)
Supplement: Supplementary file 1 — Supplementary Information [file 41598_2017_6032_MOESM1_ESM.pdf]

**A three miRNAs signature predicts survival in cervical cancer using bioinformatics analysis**

Bin LIANG , Yunhui LI , Tianjiao Wang

Legends:

Figure S1. Hierarchical clustering of cervical cancer and non-cervical cancer by differentially expressed miRNAs. The heatmap consist of 3 normal tissues (left part) and 251 cervical cancer tissues (right part). Each row represents the expression level of a miRNA, and each column represents a sample.

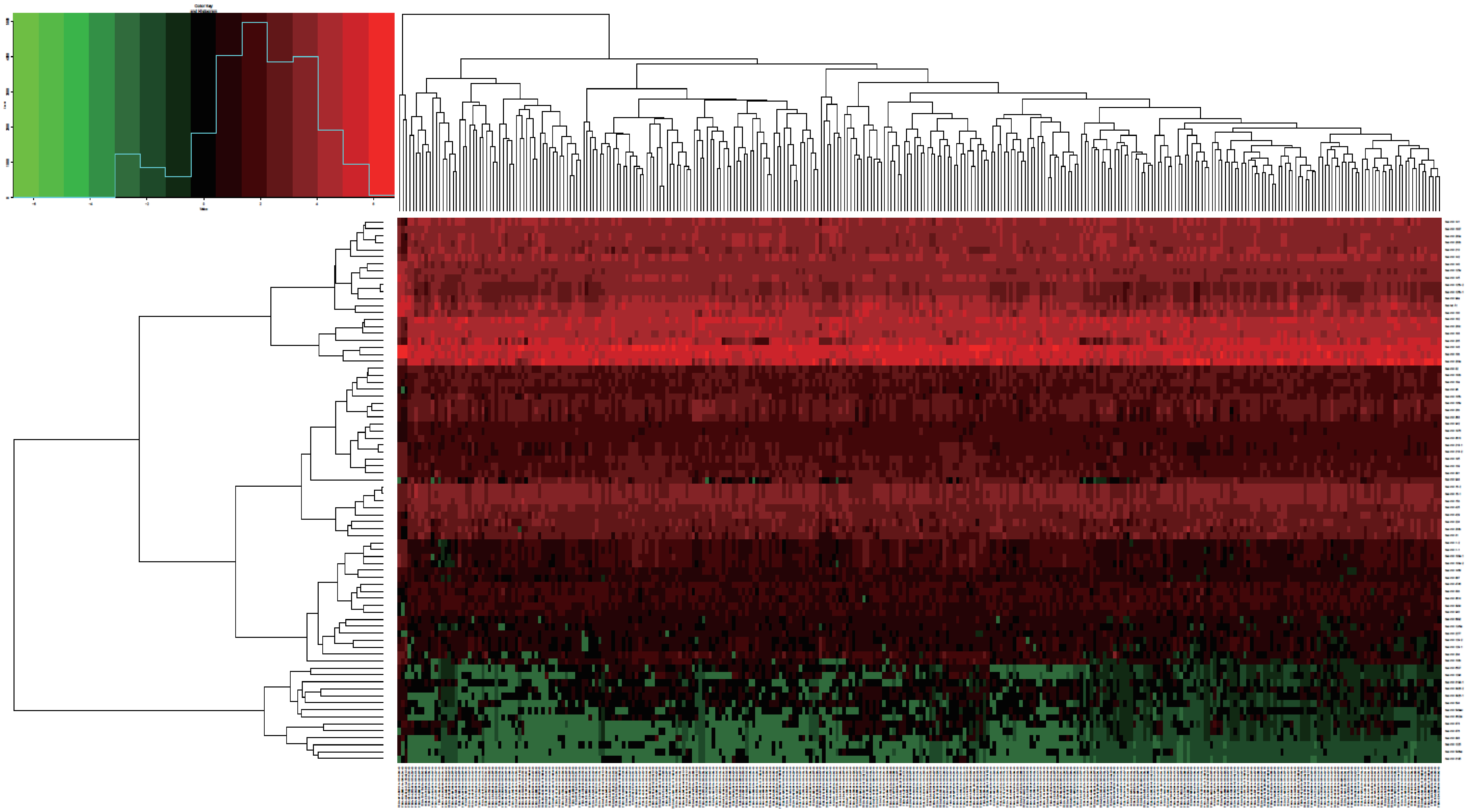

Figure S1. Hierarchical clustering of cervical cancer and non-cervical cancer by differentially expressed miRNAs. The heatmap consist of 3 normal tissues (left part) and 251 cervical cancer tissues (right part). Each row represents the expression level of a miRNA, and each column represents a sample.

**Table S1. Differentially expressed miRNAs between cervical cancer tissues and normal tissues**

| Down regulated miRNA | | | | Up-regulated miRNA | | | |
|---|---|---|---|---|---|---|---|
| miRNAs | logFC | *P* Value | FDR | miRNAs | logFC | *P* Value | FDR |
| miR-133a-2 | -4.65 | 3.66E-19 | 1.15E-16 | miR-1307 | 2.08 | 3.34E-05 | 0.0010 |
| miR-133a-1 | -4.62 | 1.25E-20 | 4.93E-18 | miR-425 | 2.09 | 0.0026 | 0.0394 |
| miR-145 | -4.33 | 1.95E-32 | 1.54E-29 | miR-16-2 | 2.12 | 0.0001 | 0.0026 |
| miR-204 | -4.19 | 6.25E-09 | 5.80E-07 | miR-16-1 | 2.13 | 0.0001 | 0.0026 |
| miR-1-1 | -4.16 | 5.18E-17 | 1.36E-14 | miR-32 | 2.18 | 3.90E-05 | 0.0011 |
| miR-1-2 | -4.15 | 1.02E-16 | 2.29E-14 | miR-1976 | 2.41 | 1.54E-05 | 0.0005 |
| miR-129-2 | -4.11 | 4.58E-13 | 7.22E-11 | miR-15b | 2.55 | 8.84E-06 | 0.0003 |
| miR-129-1 | -4.09 | 1.60E-12 | 2.29E-10 | miR-942 | 2.61 | 2.68E-05 | 0.0008 |
| miR-133b | -3.95 | 4.40E-10 | 5.33E-08 | miR-3613 | 2.63 | 2.27E-05 | 0.0007 |
| miR-873 | -3.89 | 8.16E-06 | 0.0003 | miR-4746 | 2.90 | 0.000372 | 0.0074 |
| miR-10b | -3.87 | 2.90E-34 | 4.57E-31 | miR-200b | 3.00 | 0.000491 | 0.0094 |
| miR-383 | -3.83 | 0.0004 | 0.0081 | miR-130b | 3.09 | 4.36E-06 | 0.0002 |
| miR-100 | -3.51 | 1.34E-10 | 1.76E-08 | miR-18a | 3.12 | 0.0001 | 0.0028 |
| miR-1298 | -3.40 | 0.0010 | 0.0173 | miR-940 | 3.30 | 0.0003 | 0.0069 |
| miR-3622a | -3.37 | 1.37E-06 | 6.96E-05 | miR-2277 | 3.51 | 0.00071 | 0.0131 |
| miR-143 | -3.29 | 1.94E-15 | 3.82E-13 | miR-142 | 3.55 | 7.13E-05 | 0.0019 |
| miR-6507 | -3.24 | 1.27E-05 | 0.0004 | miR-224 | 3.57 | 0.0020 | 0.0318 |
| miR-504 | -3.20 | 1.98E-07 | 1.25E-05 | miR-106a | 3.58 | 0.0013 | 0.0219 |
| miR-3195 | -3.14 | 0.0002 | 0.0038 | miR-33b | 3.61 | 0.0006 | 0.0110 |
| miR-548ba | -3.10 | 4.38E-05 | 0.0012 | miR-3934 | 3.68 | 7.06E-06 | 0.0003 |
| miR-99a | -3.09 | 7.33E-07 | 4.13E-05 | miR-3614 | 3.74 | 0.0010 | 0.0173 |
| miR-876 | -3.05 | 0.0016 | 0.0261 | miR-200a | 3.96 | 6.03E-06 | 0.00025 |
| miR-140 | -2.82 | 5.78E-24 | 3.04E-21 | miR-135b | 3.97 | 0.0006 | 0.0110 |
| miR-139 | -2.80 | 2.02E-13 | 3.53E-11 | miR-363 | 4.05 | 0.0020 | 0.0318 |
| miR-1225 | -2.80 | 0.0002 | 0.0045 | miR-429 | 4.10 | 1.11E-05 | 0.0004 |
| miR-125b-1 | -2.64 | 2.32E-08 | 1.74E-06 | miR-200c | 4.38 | 1.89E-08 | 1.57E-06 |
| miR-125b-2 | -2.57 | 1.76E-07 | 1.16E-05 | miR-182 | 4.47 | 5.54E-06 | 0.000236 |
| miR-3926-2 | -2.53 | 6.57E-06 | 0.0003 | miR-20b | 4.49 | 0.0029 | 0.043875 |
| let-7c | -2.50 | 0.0002 | 0.0044 | miR-210 | 4.85 | 2.83E-05 | 0.000857 |
| miR-548aw | -2.49 | 2.31E-06 | 0.0001 | miR-203b | 4.92 | 0.0003 | 0.00555 |
| miR-381 | -2.39 | 2.97E-07 | 1.80E-05 | miR-141 | 5.05 | 1.21E-09 | 1.28E-07 |
| miR-3926-1 | -2.39 | 0.0001 | 0.0026 | miR-183 | 5.47 | 6.03E-07 | 3.52E-05 |
| miR-6892 | -2.38 | 3.65E-08 | 2.62E-06 | miR-96 | 5.53 | 9.86E-08 | 6.76E-06 |
| miR-1468 | -2.36 | 2.13E-08 | 1.68E-06 | miR-31 | 5.60 | 0.0001 | 0.0026 |
| miR-195 | -2.21 | 1.41E-06 | 6.96E-05 | miR-203a | 5.94 | 0.0003 | 0.0061 |
| miR-1245a | -2.13 | 3.96E-05 | 0.0011 | miR-205 | 7.76 | 1.95E-05 | 0.0006 |
| miR-887 | -2.11 | 3.74E-06 | 0.0002 | miR-944 | 7.96 | 0.0009 | 0.0158 |
| miR-125a | -2.11 | 4.98E-10 | 5.61E-08 | | | | |
| miR-218-1 | -2.10 | 0.0001 | 0.0030 | | | | |
| miR-218-2 | -2.09 | 0.0001 | 0.0026 | | | | |
| miR-3199-1 | -2.08 | 7.33E-05 | 0.0020 | | | | |
